# Clinical Presentation, Bacteriologic Findings and Possible Risk Factors for Ischemic Teat Necrosis in Cattle—A Case Series

**DOI:** 10.3390/vetsci11060271

**Published:** 2024-06-14

**Authors:** Jan Kortstegge, Yanchao Zhang, Franziska Preine, Volker Krömker

**Affiliations:** 1 Department of Microbiology, Faculty II, Hannover University of Applied Sciences and Arts, 30453 Hannover, Germany; jan.kortstegge@tiho-hannover.de (J.K.); yanchao.zhang@hs-hannover.de (Y.Z.); franziska.preine@hs-hannover.de (F.P.); 2 Department of Veterinary and Animal Sciences, Section for Production, Nutrition and Health, Faculty of Health and Medical Sciences, University of Copenhagen, 1870 Frederiksberg C, Denmark

**Keywords:** bovine, dairy, veterinary medicine, udder health, ischemic teat necrosis, *Treponema* spp., *Staphylococcus aureus*, etiology

## Abstract

**Simple Summary:**

This study addresses ischemic, i.e., oxygen-deprivation-induced, teat necrosis in dairy cows, a disease that causes significant economic and animal welfare problems in the dairy industry. The aim was to investigate the clinical presentation, possible risk factors, and microbial involvement in teat necrosis. The necrosis can be recognized by an initial reddish-bloody change from the teat to the base of the udder and the eventual development of dead, dark areas and even loss of the teat, usually caused by licking by the cow. In addition to the visual change, severe itching is a particularly noticeable clinical sign. The researchers analyzed milk and swab samples from affected cows and collected questionnaires from farmers and veterinarians. The study revealed that ischemic teat necrosis predominantly affects first- and early-lactation heifers and identified infectious pathogens such as *Treponema* spp. and *Staphylococcus aureus* as well as non-infectious factors such as pre-damaged, chapped, dry, or thickened teats and certain milking factors as contributors. The results suggest that the necrosis has a multifactorial cause, combining both infectious and non-infectious factors. Understanding these factors is critical to the development of prevention and treatment strategies that aim to reduce the economic impact and improve animal welfare.

**Abstract:**

Ischemic teat necrosis (ITN) is a growing problem in the dairy industry characterized by teat lesions, necrosis, pruritus and automutilation. Despite the economic and welfare consequences, there is no treatment, and the etiology of the disease remains poorly understood. The aim of this study was to investigate ITN by analyzing its clinical presentation, potential risk factors and microbial involvement. Methods included collection of milk and swab samples from affected cows over a period of one-and-a-half years and completion of questionnaires by veterinarians and farmers. Microbial testing included PCR testing for *Treponema* spp. and cultural testing by anaerobic and aerobic incubation on blood agar. The results showed a high and significant prevalence of *Treponema* spp. and *Staphylococcus aureus* in affected teats compared to non-ITN-affected control teats, indicating their potential role in the development of ITN. Other factors such as edema and milking practices also appear to contribute to the tissue damage. First-lactation and early-lactation heifers are particularly at risk. In conclusion, ITN appears to have a multifactorial etiology with both infectious and non-infectious factors playing a role. Further research is needed to better understand the complex interplay of these factors and to develop effective prevention and management strategies.

## 1. Introduction

In general, ischemic necrosis is an irreversible cellular dysfunction caused by an extreme lack of oxygen, which is usually due to a reduction in tissue perfusion [1].

Ischemic teat necrosis (ITN) is an emerging and increasingly prevalent disease complex associated with sporadic teat lesions, necrosis, severe pruritus, and automutilation. No treatment has been described for this disease, which is associated with high economic losses and animal welfare problems [2,3]. The etiology and epidemiology thereof are largely unknown, as only a few studies are currently available. However, several different risk factors have been described and suggested in the literature.

In ITN, purulent to eosinophilic, ulcerative, and epidermal hyperplasia with dyskeratosis have been described. Leukocytoclastic to eosinophilic vasculitis and thrombosis have also been observed in some cases [2]. It appears that the severe inflammation causes severe itching, which causes cows to further damage their teats, but an exact pathogenesis has not been described in the literature.

The co-occurrence of udder cleft dermatitis (UCD), digital dermatitis (DD), or chapped teats are discussed as possible risk factors. Several rare mastitis pathogens are considered to be etiologically relevant. It has been described that first-lactation cows in early lactation are particularly affected [3,4,5].

The necrosis may extend only to the teat, or to the teat and udder. Typically, the damage progresses to the point where the affected teat with the aid of the cow is lost through automutilation. The cow is usually culled. However, in some cases, improvement or self-cure has been reported [2,3].

*Treponema* spp., *Staphylococcus* (*S.*) *aureus*, *Trueperella* (*T.*) *pyogenes,* and *Fusobacterium* (*F.*) *necrophorum* have been described as possible etiologic factors. In particular, *Treponema* spp. causing digital dermatitis have been considered a risk factor [3,4,5,6]. In a very recent study by Crosbi-Durrany et al. [6], *Treponema* spp. were detected in 35.8% of cases in 95 teats with ITN. In the control samples, *Treponema* spp. were detected in only one sample (5.6%). Thus, it was shown that *Treponema* spp. may be a factor in the development of ITN. However, the authors concluded that *Treponema* spp. were only found in some of the samples and therefore cannot be considered the most important or sole etiologic factor [6]. A survey from Germany in 2016/2017 reported that several animals on two farms were affected by ITN, particularly in the first lactation and at the beginning of lactation. Skin samples from these animals were examined and *Treponema* spp. were detected. In addition, significant peripartum udder edema was found in the affected animals, making this a significant risk factor for discussion [7]. There are reports that *Treponema* spp. also play a role in UCD [8,9]. Therefore, transmission or involvement of the pathogen in diseases of the udder is conceivable.

As the disease seems to occur predominantly in heifers at the beginning of lactation and various risk factors are discussed [3,4,5,6,7], both non-infectious and infectious factors appear to play a role in the development of ITN.

Due to the sporadic nature of the disease, which only occurs in isolated cases, systematic evidence-based studies are not available and are currently not possible. Therefore, the first step in understanding the disease is to collect and analyze occurring cases of ITN and related data. In addition, it is not possible to obtain an accurate overview of the incidence of ITN, as no studies of ITN incidence are currently available.

The aim of this study is to provide a more detailed overview of this disease complex, particularly with regard to the clinical presentation, possible risk factors and a broad overview of the possible bacterial microorganisms involved. This study is the first in Germany to examine a large number of samples from quarters affected by ITN and to classify many possible risk factors for the development of ITN using a questionnaire. In addition, for the first time, all pathogens present were identified by blood agar and MALDI-TOF analysis. In the absence of evidence-based studies on risk factors and etiologic causes, a combination of factors must be assumed based on current knowledge. It appears that both non-infectious and infectious factors play a role in the development of ITN.

With the knowledge gained from the present study, further evidence-based studies can be conducted to prove the etiology and pathogenesis of ITN. Ultimately, the goal must be to develop a treatment that minimizes the damage caused by this disease.

## 2. Materials and Methods

All applicable guidelines for the care and use of animals were followed. The study was approved by the Animal Welfare Committee of the university (University of Veterinary Medicine Hannover, Foundation, Hannover, Germany; file reference: TVO-2022-V-56). The date when ethical approval was obtained was 29 August 2022. An application for a license for animal testing was not required by the local government due to the study design. The study complied with the International Guiding Principles for Biomedical Research Involving Animals (1985).

### 2.1. Sampling of Milk and Swab Samples

To obtain milk and swab samples, an appeal was made at national conferences throughout Germany and via important German agricultural journals to report any cases that occurred. The farms were asked to contact the University of Applied Sciences and Arts Hannover, Hannover, Germany or the corresponding author (V.K.) if the described symptoms were observed. In the presence of ITN, the attending veterinarians or farmers were asked to collect milk samples from the affected cow and swab samples from the interface between the affected area and the healthy udder skin. Instructions were given on how to sample and missing materials were sent when required. For swab sampling, some material should be removed from the undisinfected teat at the transition from lesion to unaffected skin using light pressure so that material was visible on the swab. For milk samples, the teats should be carefully cleaned before sampling and then milked into a milk sample tube so that no contaminants can enter the tube. The aseptic collection of milk samples should be performed according to GVA (2018) [10], and the swab sampling modified according to DIN standard: 10113-1:1997:07, but with only one swab. The samples were then sent to the Microbiology lab at the University of Applied Sciences and Arts Hannover for testing within 48 h. Samples were collected over a period of around one-and-a-half years (November 2022 to March 2024) and stored frozen at −20 °C until analysis in February and March 2024.

A total of 23 farms provided samples of udder quarters with ITN. Samples were collected from 35 different cows. If more than one quarter was affected, all affected quarters were sampled. In total, samples were collected from 50 affected quarters. In addition, milk samples were available from 28 non-affected quarters that were collected in addition to the affected quarters. As milk could not always be milked from the udder quarter, only swab samples were available from a total of nine quarters. On two farms, it was not possible to collect a swab sample because the material was not available before the cow was removed from the farm. In summary, of the 50 affected teats, both milk and swab samples were available for 39 teats, while only one swab sample was available for 9 teats and only one milk sample was available for 2 teats.

### 2.2. Data Sampling

To obtain a more precise overview of the symptoms of the disease and possible risk factors, the attending veterinarians and farmers were asked to complete a questionnaire. The questionnaire asked for general farm information (number of cows, type of milking, milking vacuum level, etc.), details of the cow’s history and clinical examination of cow and udder, and whether there were any other udder or hoof diseases on the farm (e.g., DD, UCD, mastitis) associated with the disease (see Appendix A). The completion of the questionnaire was done on a voluntary basis. Of the 23 farms that provided samples, 15 responded to the questionnaire. For the remaining eight farms, information is only available in a few cases, e.g., through e-mail correspondence. In addition, two farms whose cows were removed from the farm before sampling completed the questionnaire so that they could be included in the study. In total, 25 farms with at least one affected animal responded to the questionnaire. Completed questionnaires were available for a total of 34 ITN-affected quarters. For 18 quarters, no questionnaires were returned. As the completed questionnaires varied in detail and it was not mandatory for veterinarians and farmers to answer all items, only the items that were answered can be considered. The most important information provided in the questionnaires is shown in Table 1.

### 2.3. Preparation of the Swab Samples and Cultural Examination on Blood Agar

To distribute the swab samples on blood agar, they were vortexed in 500 μL Ringer’s solution for at least one minute. Subsequently, 200 μL of this solution could be used for culture and the remaining solution for PCR testing.

To obtain a complete overview of possible pathogens, culture was performed on blood agar. The test was modified according to the GVA guidelines (2018) [10]. As a qualitative test for all possible pathogens was to be performed, 100 μL of each milk sample and of the swab sample solution were transferred to an entire blood agar plate and the plates were examined several times to increase diagnostic accuracy. The plates were incubated anaerobically and aerobically at 37 °C. For anaerobic incubation, a gel was used that ensures an O_2_ concentration of <0.1% within 2.5 h and a CO_2_ concentration of 7–15% within 24 h (Oxoid Ltd., AnaeroGen, Wesel, Germany). Morphologically different grown colonies were isolated on new blood agar plates and incubated. The aerobically incubated plates were examined after 24, 48 and 72 h and the colonies grown were determined by Matrix-assisted laser desorption/ionization time-of-flight (MALDI-TOF). For the anaerobically incubated plates, these determinations were carried out after 24 and 72 h as well as after 7 days. For each udder quarter, pathogen detection was evaluated during aerobic and anaerobic incubation and for milk and swab samples combined.

### 2.4. PCR Testing for Treponema spp.

A PCR test was performed to examine the samples for the presence of *Treponema* spp. Both 300 μL of the milk samples and 300 μL of the swab sample solution (see Section 2.3) were used for DNA extraction. For chromosomal DNA extraction, the DNeasy Blood and Tissue Kit from Qiagen GmbH, Hilden, Germany was used, and PCR was performed according to previously published methods. The primers used (forward primer 5′-CAAGGCDWYGATGGGTAT-3′, reverse primer 5′-GTCAGACTTYCGTCCATTG-3′) and PCR conditions with an initial activation of ten minutes at 95 °C, followed by 40 cycles of one minute at 95 °C and one minute at 60 °C with a final cycle for the generation of a dissociation curve of one minute at 95 °C, followed by 30 s at 55 °C and 30 s at 95 °C, were based on previous studies with slight modifications [11]. For each PCR run, negative controls (H_2_O) and positive controls of treponemes from DD cases from the laboratory’s pathogen database were also examined and the melting curves were compared with the positive samples. In cases of uncertainty, additional gel electrophoresis was performed and compared to the positive controls.

### 2.5. Statistical Analysis

To verify the prevalence of the pathogens analyzed, a chi-square (X^2^) test was performed to calculate the prevalence of the pathogens comparing ITN-affected and non-ITN-affected udder quarters. A prevalence of <0.05 was defined as significant.

## 3. Results

### 3.1. PCR Testing for Treponema spp.

In the PCR test for *Treponema* spp., 38 (76%) of the 50 samples sent from the affected quarters were positive for *Treponema* spp. (Table 2). In eight cases, both the milk and swab samples were positive. In twenty-four of the thirty-eight positive cases, only the swab sample was positive, and in six cases only the milk sample was positive. Of the twenty-four milk samples sent in from teats not infected with ITN, only one (4.2%) sample was PCR-positive for *Treponema* spp. (Table 2).

### 3.2. Results of the Cultural Incubation

Of the 50 ITN quarters, *S. aureus* was detected on blood agar in samples from 33 (66%) different quarters. Nine of the thirty-three quarters also tested positive for *Treponema* spp. In 24 (48%) cases, there was growth of *S. aureus* while testing for *Treponema* spp. was negative. In the samples not affected by ITN, four of twenty-four (16.7%) milk samples showed growth of *S. aureus* on blood agar (Table 2).

*T. pyogenes* was detected in samples from six quarters (12%). In all six cases, the udder quarters were also positive for *S. aureus* (*n* = 4) and/or *Treponema* spp. (*n* = 4).

*Streptococcus (Sc.) dysgalactiae* growth on blood agar was present in 26 (52%) of the 50 udder quarters affected by ITN. Two of the positive *Sc. dysgalactiae* quarters were negative for both *S. aureus* and *Treponema* spp. Of the twenty-four control quarters not affected by ITN, six (25%) were positive for *Sc. dysgalactiae*.

In addition, 11 (22%) of the 50 affected ITN quarters were positive for *Sc. uberis*. All 11 quarters were positive for either *S. aureus* or *Treponema* spp. In quarters not affected by ITN, *Sc. uberis* growth was present in two (8.3%) of the twenty-four quarters.

In total, 47 (94%) of the ITN-affected quarters tested positive for at least *S. aureus* or *Treponema* spp. Only three (6%) samples were negative for both *Treponema* spp. and *S. aureus*. However, two of these samples showed growth of *Sc. dysgalactiae*. Thus, one (2%) sample did not show either a positive PCR result for *Treponema* spp. or growth of *S. aureus* or *Sc. dysgalactiae* on blood agar. Nonetheless, growth of *Escherichia* (*E.*) *coli* on blood agar was detected in this case.

In the quarters not affected by ITN, a total of 19 of 24 (79.2%) quarters were negative for both *Treponema* spp. and *S. aureus*. In all, 13 (54.2%) of these 24 quarters were additionally negative for *Sc. dysgalactiae* and *T. pyogenes*.

In addition, non-aureus staphylococci (NaS) (41/50 udder quarters positive; 82%), *Corynebacterium* spp. (3/50; 6%), *E. coli* and coliforms (12/50; 24%), or enterococci (18/50; 36%) were present in some samples of the quarters affected by ITN.

### 3.3. Results of the Questionnaire

#### 3.3.1. Clinical Symptoms and Pre-Existing Conditions

**Figure 1:**
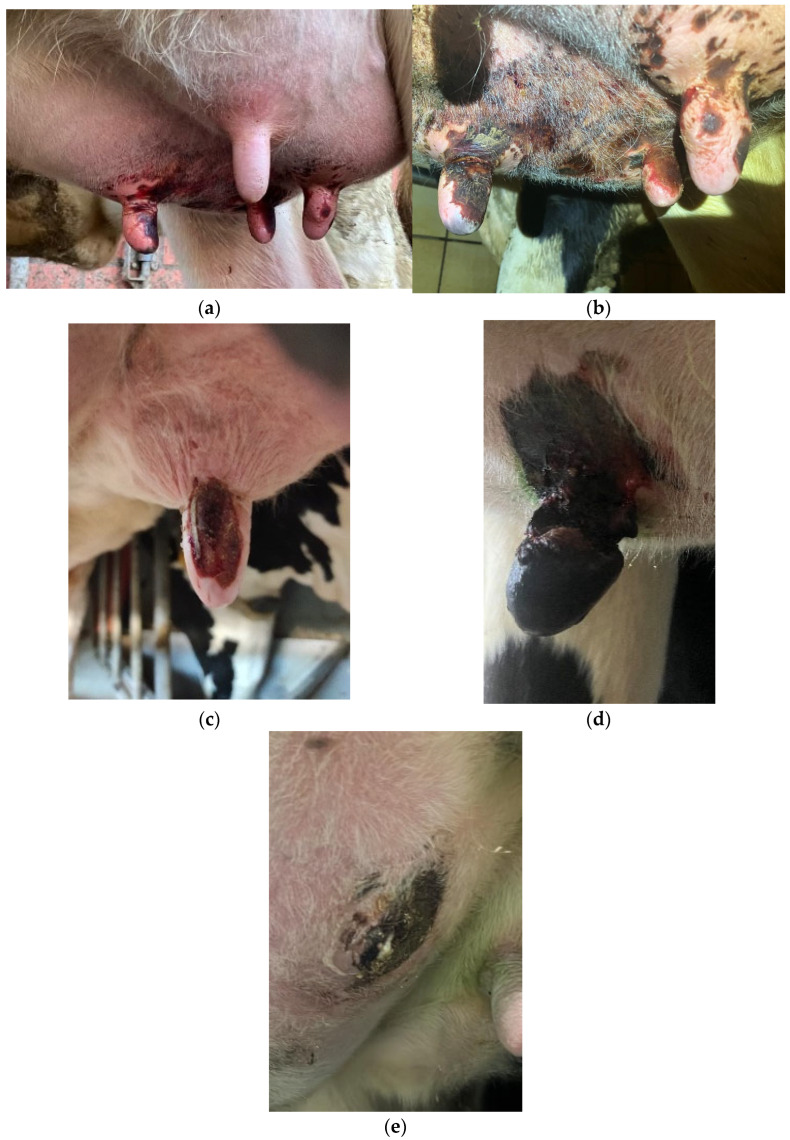
(**a**–**e**). Ischemic teat necrosis (ITN), teat and udder. (**a**,**b**) Two cows with ITN from one farm—(**a**) early stage and (**b**) more advanced stage with clear blackish discoloration. (**c**) Bloody crusty change limited to the teat. (**d**) Completely dark discolored teat with significant depth injury. (**e**) Residual necrosis on udder without teat—teat completely detached from udder. The images were provided by the farmers or veterinarians who participated in the study: (**a**,**b**) Anonymous, (**c**) Anonymous, (**d**,**e**) Mrs. Schäfer-Wolf.

A form of necrosis was described in all cases. Descriptions of the appearance and extent of symptoms ranged from “bloody crusty” to “bluish discoloration with skin sloughing” (Figure 1a–c). The progression was described as an “initial reddish-bluish discoloration that became black with time”. In another case, the progression was described as “initially red and sore, later hard and scabby to the point of rupture”. The necrosis and lesion progressed to the interior of the teat (Figure 1d). The bloody spots were mainly caused by self-licking or kicking by the cow. Only a “bleeding udder without teat” was reported in one case where the cow died before sampling.

The extent of the lesions varied widely in the reports. In some cases, the lesions were limited to the tip or middle of the teat. However, there have also been reports of the lesion being located at the attachment of the teat to the base of the udder or extending over the entire teat and base of the udder. In many cases, the teat finally separated from the udder (Figure 1e).

As can be seen in Table 3, various symptoms and external changes of the teat were recorded in the questionnaires. The most reported symptoms were automutilation and pruritus. Different degrees of severity were described. In one case, automutilation was rated as mild. In other cases, very severe automutilation or automutilation resulting in “licking away” of the teats was reported. In most cases, automutilation was detected by the cow licking herself, but kicking with the hind legs was also possible.

In 15 of 34 (44.1%) quarters with ITN, it was described that the teat was particularly dry. One farm noted that there was a high level of lime in the cow’s lying area, which was thought to be the cause. However, in another questionnaire, the teat was described as looking exudative rather than dry.

Thickened and chapped teats were observed in 11 (32.4%) cases each. Other individually mentioned abnormalities included the presence of wounds, in some cases just prior to the development of necrosis, and skin sloughing.

The most common symptom preceding necrosis was described as udder and teat edema (Table 3). According to the data provided, the severity varied from “some” or “mild” to “more than usual”, “severe”, and “especially in heifers”.

DD was only sporadically observed in the affected cows. In some cases, there was mention of a problem with DD in the herd. However, the majority of questionnaires did not report any problems with DD.

Mastitis was another rarely reported pre-existing condition. One report described the occurrence of mastitis caused by *Sc. uberis*. In this case, however, the mastitis was thought to be secondary to the ITN. In another case, mastitis caused by *S. aureus* was reported to be present three days after calving prior to the occurrence of ITN.

#### 3.3.2. Potential Animal Factors of ITN

A total of 35 of the affected teats came from Holstein Friesian cows (Table 4), or in one case, a mixture of Holstein Friesian (HF) and Montbéliarde. This represents 92.1% of known cases (*n* = 38). Three of the quarters (7.9%) were from Brown Swiss cows. The breed of the remaining cows is unknown.

In most cases (Table 4), cows in their first lactation (72.7%) at the start of lactation (82.2%) were affected. However, there were isolated reports of ITN in cows in their second or later lactation. For example, eight quarters affected by ITN were from cows in their fourth or seventh lactation. There were also some reports of cows in later stages of lactation being affected. Two cases were reported where ITN was evident at 240 and 290 DIM. In one case, ITN problems were reported to have been present since the beginning of lactation and were still present at 250 DIM.

#### 3.3.3. Farm-Specific and Milking Factors on Farms with ITN Outbreaks

The size of the farms in which ITN cases occurred showed a wide range according to the data received (average 219 cows). Smaller farms with 21 or 70 cows were represented in the study as well as larger farms with 660 or 880 cows.

The milking technique also showed a balanced ratio (Table 5). A total of 21 (52.5%) of the udder quarters affected by ITN came from farms with conventional parlor milking and 16 (40%) from farms with an automatic milking system (AMS). One farm reported that it had changed from a parlor to an AMS only three months before the first occurrence of ITN in the herd.

Agents based on lactic acid, iodine, or chlorhexidine were used for the dipping of the teats. Peracetic acid products were mainly used for intermediate disinfection, although it was also reported that intermediate disinfection was not performed or was performed only on cows with elevated somatic cell counts in the milk.

When asked if this was the first time ITN had been detected in the herd, this was confirmed on several occasions (*n* = 21). In one case, the disease appeared for the first time, but eight cows were infected simultaneously. In other reports, ITN had been observed sporadically (*n* = 3) or several times (*n* = 6) in the past.

There were isolated anecdotal reports in the questionnaires listed under other observations. One farm reported an improvement in necrosis after application of an udder net or zinc spray. Another farm reported a worsening of symptoms after application of Vaseline or certain other ointments. In addition, one veterinarian stated that he suspected a link to poor grass silage, which may have led to increased teat edema in heifers. On one farm, ITN cases were thought to be due to the change from a milking parlor to an AMS, as the change occurred three months before the first case of ITN.

## 4. Discussion

### 4.1. Clinical Appearance of the Disease

The clinical picture and the symptoms seen in the cases included in this study are consistent with the descriptions in earlier reports [2,3,4,5]. The teats affected by ITN most frequently showed pruritus, automutilation by the cow, and dry, chapped, and thickened teats. It should be noted that it is often not clear from the questionnaires whether the symptoms were present prior to the development of necrosis or whether they were part of the disease picture. For example, chapped and thickened teats were considered a significant risk factor.

Based on the data presented, it seems clear that young cows, usually in their first lactation, are a risk group. In addition, animals early in lactation, usually <100 DIM, are particularly affected by ITN. These findings are also in accordance with previous studies [3]. However, the results presented also shows that older cows and cows in later stages of lactation can also be affected and that the focus of further studies should not only be on the first group of young cows.

### 4.2. Possible Etiologic Causes

The aim of this study was to obtain the broadest possible overview of pathogenic bacteria that may be involved in the development of ITN. Therefore, mesophilic anaerobic and aerobic microorganisms were grown on a blood agar. This will at least identify the bacteria that can be therapeutically influenced at this time. The blood agar test was carried out over several days in order to cultivate even slow-growing microorganisms. The actual identification was done by MALDI-TOF. All grown pathogens were isolated and identified by MALDI-TOF. PCR testing for *Treponema* spp. was performed, as *Treponema* spp. are the most discussed factor in the existing literature [3,4,5,6]. However, detection of *Treponema* spp. by culture is very difficult [12], so PCR testing for *Treponema* spp. was performed.

In this study, the pathogens present in the ITN-infected teats were examined in both milk and swab samples. It is noteworthy that all but three teats were infected with *Treponema* spp. and/or *S. aureus*. *Treponema* spp. in particular have been previously associated with the occurrence of ITN and discussed as a possible etiologic cause [3,4]. A 2010 article discussed the role of *Treponema* spp. in the development of bovine ulcerative dermatitis. It was concluded that although *Treponema* spp. play a role, it is more of a polymicrobial event and *Treponema* spp. are not the main or only cause [9]. Our study on ITN shows a similar picture. As 76% of the teats affected by ITN tested positive for *Treponema* spp., this study reveals that *Treponema* spp. are a possible etiologic factor. Univariable significance was demonstrated (Chi Square test; *p* < 0.00001). However, secondary infection with *Treponema* spp. must be considered in addition to non-infectious causes and other pathogens that can cause necrosis. Crosby-Durrani et al. described in their study that they considered that milk and foremilk are not a viable reservoir of infection for DD-associated *Treponema* spp. and that the detection of *Treponema* spp. in milk is unlikely [6]. In contrast, a study by Sobhy et al. described good detection of *Treponema* spp. in foremilk and even considered foremilk as a possible transmission route for DD-associated *Treponema* spp. [13]. As *Treponema* spp. were detected in both milk and swab samples in our study, detection in both materials appears to be possible. It should be noted that the PCR assay only identifies *Treponema* spp. and no primers for specific pathogenic *Treponema* species were used. Therefore, possible contamination with non-pathogenic *Treponema* species must be considered. However, positive controls of DD treponemes were also analyzed in the PCR and the dissociation curves were compared. In addition, *Treponema* spp. were detected much more frequently in the milk samples from the ITN affected quarters than in the negative control samples. Nevertheless, in 24 of 36 cases, *Treponema* spp. were detected only in the swab sample, so detection in the swab appears to be more sensitive. Since *Treponema* spp. were reported in only eight (16%) of the fifty cases on the farm or in the corresponding cow, the question arises as to the origin of the *Treponema* spp. detected in the samples from the ITN teats. Whether these were *Treponema* spp. that also caused DD cannot therefore be adequately clarified, even if the data from previous studies indicate this [4].

*S. aureus*, with a prevalence of 66%, must also be discussed as an important etiologic factor (<0.001). Although the literature on skin disease caused by *S. aureus* in cattle is not very extensive, its occurrence is described in the disease known as impetigo, in which the changes can spread to the udder skin and teat base and are associated with swelling and edema [14].

Another skin disease of the udder is called udder dermatitis. It is characterized by trauma to the skin, followed by edema and swelling after parturition, on the side of the udder or in another form in the intermammary fold. In this disease, pressure also causes extensive necrosis, ulceration, and keratinous debris. Various pathogens are discussed as the main etiologic cause of this disease. These include *F. necrophorum* and *T. pyogenes* [14]. *F. necrophorum* was not detected in this study, so a primary role in ITN is unlikely. However, assuming a multifactorial process in which necrosis-inducing pathogens have a secondary effect, it is possible that *F. necrophorum* may contribute to the development or aggravation of necrosis in other cases. Therefore, the involvement of *F. necrophorum* should be further monitored. *T. pyogenes* was detected in 12% of the teats with ITN, so it is possible that it was involved in these cases. However, in all six cases, either *S. aureus* or *Treponema* spp. were also positive, so *T. pyogenes* should rather be considered as a potentiating infection.

Overall, the presence of bacteria appears to be a possible etiologic factor (*p* < 0.05), but since both *S. aureus* and *Treponema* spp. were not always detected, this suggests that ITN is a multifactorial event in which non-infectious factors must be present in addition to infectious causes. This conclusion is also consistent with a recent study from the United Kingdom [6]. The fact that in many cases only individual teats or single animals in the herd were affected, and that farms have reported no further cases for a long time after the onset of ITN, indicates that it is most likely not a contagious event or that non-infectious factors must necessarily be present. It can therefore be assumed that, in cases where several animals were affected at the same time, there is a general problem with milking management, feeding or other factors (see non-infectious causes). Bacterial infection may be secondary and may trigger or exacerbate the disease.

Colonization with mastitis-causing pathogens such as *Sc. dysgalactiae* or *Sc. uberis* is also likely to occur secondarily after preliminary teat damage has occurred. The development of mastitis and the associated inflammatory response can thus further exacerbate the ITN.

It should also be noted that viral pathogens may also play a role in the development of ITN. This has not been investigated due to financial and laboratory capacity limitations. However, the involvement of possible viral pathogens has also been poorly investigated in the literature. Only the involvement of bovine herpesvirus type 4 (BHV-4) was discussed in a German case study but it was considered an unlikely cause for the development of ITN [7].

### 4.3. Possible Non-Infectious Causes

The occurrence of edema was reported as the most common risk factor. Edema and enlargement or hardening of the teat can lead to pressure necrosis as the oxygen supply to the tissue is reduced. Milking, in particular, can be an aggravating factor. Milking with increased vacuum (>43 kPa) has also been considered a risk factor, as it can lead to machine-induced teat edema, increased stress on the teat tissue, and morphological changes of the teat such as thickening and elongation [15,16,17]. According to the available data, the majority of cows with ITN were not milked with increased vacuum. Therefore, based on these results, it does not appear to be a significant factor, but in the ten cases where the milking vacuum was increased, it may still have been a factor that favored or increased the development of ITN. The presence of an AMS also did not appear to be a significant risk factor for the development of ITN. The disease occurred on farms with conventional milking parlors as well as on AMS-farms.

Based on the available data, there is no clear difference in the incidence of specific dips based on iodine, lactic acid, or chlorhexidine. No significant statement can be made on whether the use of peracetic acid for intermediate disinfection favors the development of ITN.

### 4.4. Other Possible Risk Factors and Limitations of the Study

Other factors that may be important, but were not investigated in our study, are genetic factors and differences in the development of heifer and cow teats.

As the disease seems to be increasing significantly in recent years, genetic factors must be considered. However, as HF, mixed breeds with HF, and Brown Swiss were affected in our study, a breed predisposition is possible but less likely.

Due to the higher incidence in first-lactation heifers, the difference between heifer teats and those of older cows should be investigated. It is possible that the smaller teats of heifers are more vulnerable during milking and thus more likely to promote the occurrence of ITN. Other diseases of the udder skin, such as udder cleft dermatitis, are also more common in first-lactation cows [18,19]. This shows that the udders of first-lactation cows appear to be particularly sensitive. In addition, the presence of udder edema also appears to be strongly associated with the development of changes in this disease [19].

In general, it must be noted that the answers to the items in the questionnaire were based on the expertise and accuracy of the veterinarians and farmers. In the future, with a better understanding of the most important risk factors, it should be possible to ask more precise questions about these issues and to create an objective scale to compare the responses. In this context, it is particularly useful to take a closer look at factors that are usually described subjectively, such as the milking process with attachment of the milking equipment, cleaning, and the presence of blind milking. The statements on whether DD occurs in the herd were also not always clear. In particular, milking edematous or injured teats could pose a risk for the development of ITN.

In addition, further studies are needed to investigate the extent to which edema causes the occurrence of ITN and whether this is a possible starting point for preventing the development of ITN. Lactation age, DIM, pre-calving feeding, genetic factors, and others have been considered as possible risk factors for the occurrence of udder edema [18,20,21].

Anecdotal reports in the questionnaires, such as an improvement after application of an udder net or zinc spray, or a worsening after application of petroleum jelly or certain other ointments, do not have a clear burden of proof and would need to be further investigated in separate studies before their use is considered appropriate and is authorized.

## 5. Conclusions

The study investigated the clinical presentation and potential causes of ITN in cows. Symptoms such as pruritus, automutilation, and thickened teats were consistent with previous reports, with first lactation and early lactation heifers being particularly susceptible. Pathogen analysis revealed a high and significant prevalence of *Treponema* spp. and *S. aureus* in infected teats, suggesting their potential role in the development of ITN. However, these pathogens were not found in all quarters affected by ITN. Therefore, our study suggests a multifactorial etiology for ITN, with non-infectious factors such as edema, milking practices, etc. also contributing to tissue damage. Further research is warranted to clarify the complex interaction between infectious and non-infectious factors in ITN development and to target prevention and management strategies.

## Figures and Tables

**Table 1:** Anonymized list of participating farms and presentation of key information from questionnaires and personal correspondence.

Farm	Affected Cows	Affected Quarters (Per Cow)	Number of Animals	Breed	Age of Lactation—Per Cow	DIM ^1^—Per Cow	Milking System	Milking Vacuum	Pre-Existing Conditions	Teat Conditions
1	1	1	150	HF ^2^	1	25	Both	n.I. ^3^	-	C ^4^, P ^5^, A ^6^, D ^7^
2	4	4 (1, 1, 1, 1)	660	HF	n.I.	all <100 d	MP ^8^	46	M ^9^	n.I.
3	1	1	75	BS ^10^	1	13	AMS ^11^	43	E ^12^, DD ^13^, F ^14^	C, P, A
4	1	4	n.I.	HF	7	199	AMS	n.I.	SU ^15^	C
5	5	5 (1, 1, 1, 1, 1)	120	HF	1,2,1,4,2	7, 30, 130, 240, 290	MP	n.I.	E	T ^16^ (3/5)
6	1	1	n.I.	n.I.	n.I.	n.I.	n.I.	n.I.	n.I.	n.I.
7	2	4 (2, 2)	185	HF	1,1	67, 58	AMS	41.6	E, DD	P, A, D
8	1	1	n.I.	n.I.	n.I.	n.I.	n.I.	n.I.	n.I.	n.I.
9	1	1	123	n.I.	1	<100 d	MP	42	E, DD	D
10	4	4 (1, 1, 1, 1)	240	HF	1,1,1,1	110, 25, 38, 60	AMS	44	E (one cow)	P, A, D, T (2/4)
11	1	2	100	HF	n.I.	n.I.	Both	n.I.	n.I.	n.I.
12	1	1	120	HF	1	30	AMS	44	-	P, A, D, T
13	1	1	200	HF + MB ^17^	4	8	MP	44	-	C
14	1	1	n.I.	n.I.	1	n.I.	n.I.	n.I.	n.I.	n.I.
15	1	4	n.I.	n.I.	1	<100 d	n.I.	n.I.	n.I.	n.I.
16	1	3	n.I.	n.I.	1	<100 d	n.I.	n.I.	n.I.	n.I.
17	1	1	337	HF	1	7	MP	42	E, F	C, A
18	1	2	21	BS	7	71	MP	41	M	D
19	1	1	n.I.	HF	1	20–50	MP	n.I.	F	C, P, A
20	1	1	168	n.I.	2	<100 d	MP	42	-	C, P, A, D, T
21	1	4	880	HF	1	<100 d	MP	42	E	P, A, T (1/4)
22	1	1	70	HF	1	70	MP	40.2	E, DD	C, D, T
23	2	2 (1, 1)	n.I.	HF	1	n.I.	n.I.	n.I.	n.I.	n.I.
24 *	1	1	170	n.I.	1	25	AMS	43	E, DD	P, A, T
25 *	1	1	108	n.I.	2	<50	AMS	43	E	T

^1^ Days in milk; ^2^ Holstein Friesian; ^3^ No Information; ^4^ Chapped; ^5^ Pruritus; ^6^ Automutilation; ^7^ Dry; ^8^ milking parlor; ^9^ Mastitis; ^10^ Brown Swiss; ^11^ automatic milking system; ^12^ Edema; ^13^ Dermatitis digitalis; ^14^ Fever; ^15^ Sole ulcer; ^16^ Thickened; ^17^ Montbéliarde. * No samples were available from farms 24 and 25, but a questionnaire was submitted.

**Table 2:** Results of PCR and cultural examination of ITN-affected and not ITN-affected udder quarters.

Pathogen	ITN ^1^-Affected		Not ITN-Affected		Chi Square Test
					(*p*-Value)
	positive	negative	positive	negative	
*Treponema* spp.	38/50 (76%)	12/50 (24%)	1/24 (4.2%)	23/24 (95.8%)	<0.01
*S.* ^2^ *aureus*	33/50 (66%)	17/50 (34%)	4/24 (16.7%)	20/24 (83.3%)	0.01
*T.* ^3^ *pyogenes*	6/50 (12%)	44/50 (88%)	4/24 (16.7%)	20/24 (16.7%)	0.85
*Sc.* ^4^ *dysgalactiae*	26/50 (52%)	24/50 (48%)	6/24 (25%)	18/24 (75%)	0.05
*Sc. uberis*	11/50 (22%)	39/50 (78%)	2/24 (8.3%)	22/24 (91.7%)	0.26
without *Treponema* spp. and/or *S. aureus*	3/50 (6%)	47/50 (94%)	19/24 (79.2%)	5/24 (20.8%)	<0.01
without all pathogens above	1/50 (2%)	49/50 (98%)	13/24 (54.2%)	11/24 (45.8%)	

^1^ Ischemic teat necrosis; ^2^ Staphylococcus; ^3^ Trueperella; ^4^ Streptococcus.

**Table 3:** Reported clinical symptoms and pre-existing conditions per teat and relative percentage (%) of available data for ischemic teat necrosis (ITN)-affected quarters.

**Clinical Appearance**	**Chapped Teats**	**Pruritus**	**Automutilation**	**Thickened Teats**	**Dry Teats**
	11/34 (32.4%)	18/34 (52.9%)	19/34 (55.9%)	11/34 (32.4%)	15/34 (44.1%)
**Pre-existing conditions**	**Edema**	**Digital Dermatitis**	**Mastitis**	**Fever**	
	20/34 (58.8%)	8/34 (23.5%)	6/38 (15.8%)	3/34 (8.8%)	

**Table 4:** Animal-specific factors in cows with ITN.

	Animal-Specific Factors		
**Breed**	**Holstein Friesian**	**Brown Swiss**	**No Information**
	35/52 (67.3%)	3/52 (5.8%)	14/52 (26.9%)
**Age of Lactation**	**1st Lactation**	**2nd Lactation**	**>2nd Lactation**
	32/44 (72.7%)	4/44 (9.1%)	8/44 (18.2%)
**Days in Milk**	**<100 DIM ^1^**	**100–200 DIM**	**>200 DIM**
	37/45 (82.2%)	6/45 (13.3%)	2/45 (4.5%)

^1^ Days in milk.

**Table 5:** Machine milking and milking hygiene factors on farms with ischemic teat necrosis (ITN). Prevalence and proportion of cases reported in the questionnaires compared to total data reported for ITN-affected quarters.

	Milking Related Factors		
**Milking technique**	**Milking parlor**	**Automatic milking system**	**Both ^1^**
	21/40 (52.5%)	16/40 (40%)	3/40 (7.5%)
**Milking vacuum**	**<43 kPa ^2^**	**43 kPa**	**>43 kPa**
	14/27 (51.9%)	3/27 (11.1%)	10/27 (37%)
**Basis of dipping agent ^3^**	**Lactic acid**	**Iodine**	**Chlorhexidine**
	15/33 (45.4%)	13/33 (39.4%)	5/33 (15.2%)
**Agent for intermediate disinfection**	**Peracetic acid**	**No intermediate disinfection**	
	9/24 (37.5%)	15/24 (62.5%)	

^1^ Both = on the farm, milking is performed in a milking parlor as well as in an automatic milking system; ^2^ Kilopascal; ^3^ several agents have been reported—only the main agent on which the product is based is listed.

## Data Availability

The original contributions presented in the study are included in the article, further inquiries can be directed to the corresponding author.

## Appendix A

The original questionnaire is in German. An English translation is given in parentheses.

**Protokoll—Ischemic teat necrosis**
  1. **Betriebsdaten [Farm-specific factors]**
    • Anzahl Tiere [Number of animals]:
    • durchschnittliche Milchleistung [Average milk yield]:
    • Melktechnik (konventionell, Roboter) [milking technique (conventional, automatic milking system)]:
    • Melkvakuum [Milking vacuum]:
    • Probleme mit Blindmelken?, Anmelken bei Färsen (Stimulation?, Wartezeiten bis Ansetzen des Melkgeschirrs) [Problems with blind milking?; special aspects of milking heifers?]:
    • weitere Besonderheiten beim Melken/ Melkvorbereitung [Other special aspects of milking/preparing for milking]:
    • Zitzendippmittel (v.a. bei Veränderungen in der letzten Zeit) [Teat dipping agent (especially with recent changes)]:
    • Desinfektionsmittel zur Melkzeugzwischendesinfektion [Disinfectant for intermediate disinfection]:
    • Vorbereitungsfütterung (v.a. bei Veränderungen in der letzten Zeit) [Antepartum cow feeding (especially if there have been recent changes)]:
  2. **Anamnese [Anamnesis]**
    • Einzeltiererkrankung oder Bestandsproblem bzw. bereits Fälle in der Vergangenheit? [Only one cow affected or herd problem/previous cases in the past?]:
    • Laktationsalter [Age of lactation]:
    • Laktationsstadium [Day of lactation / DIM]:
    • durchschnittliche Milchleistung des betroffenen Tieres [Average milk yield of affected cow]:
    • Euterveränderungen [Changes in the udder]:
      ○ rissige Zitzen [Chapped teats]
      ○ Juckreiz [Pruritus]
      ○ Automutilation
      ○ bekannte Klauenerkrankungen/lahme Kühe? [Known diseases of the cow]:
      ○ Geburtsprobleme [Problem at birth]:
      ○ peripartales Euterödem [Peripartum udder edema]
      ○ Fieber [Fever]
      ○ Milchleistungsminderung (Differenz) [Milk yield decrease (difference)]:
  3. **klinische Allgemeinuntersuchung [Clinical examination]**
    • Fieber [Fever]:
    • weitere Auffälligkeiten [Other abnormalities]:
  4. **spezielle US—Euter [Special examination of the udder]**
    • Lokalisation der Veränderung (Zitzenbasis?) [Localization of the change]:
    • Nekrosen [Necrosis]
    • Verdickt [Thickened]
    • Trocken [Dry]
    • Rissig [Chapped]
    • Schmerzhaftigkeit [Soreness]
    • Farbabweichung [Deviations in the color]:
    • Geruchsabweichung [Deviations in the odor]:
    • Automutilationsanzeichen („Selbstverstümmelung“) [Automutilation]:
    • weitere Auffälligkeiten [Other abnormalities]:
  5. **weitere vorliegende Erkrankungen [Other pre-existing or concomitant conditions]**
    • Dermatitis digitalis („Mortellaro“)
    • akuten interdigitale Nekrobazillose (Panaritium) [Acute interdigital necrobacillosis]:
    • UCD („Zwischeneuterdermatitis“) [Udder cleft dermatitis]
    • Mastitis (Milchprobe) [Mastitis—milk sample]:
      ○ Aktuell [Current]:
      ○ in der vorangegangenen Laktation (falls Erreger bekannt bitte angeben) [In previous lactation (please specify if pathogen known):
    • weitere aktuelle Erkrankungen [Other current diseases]:
